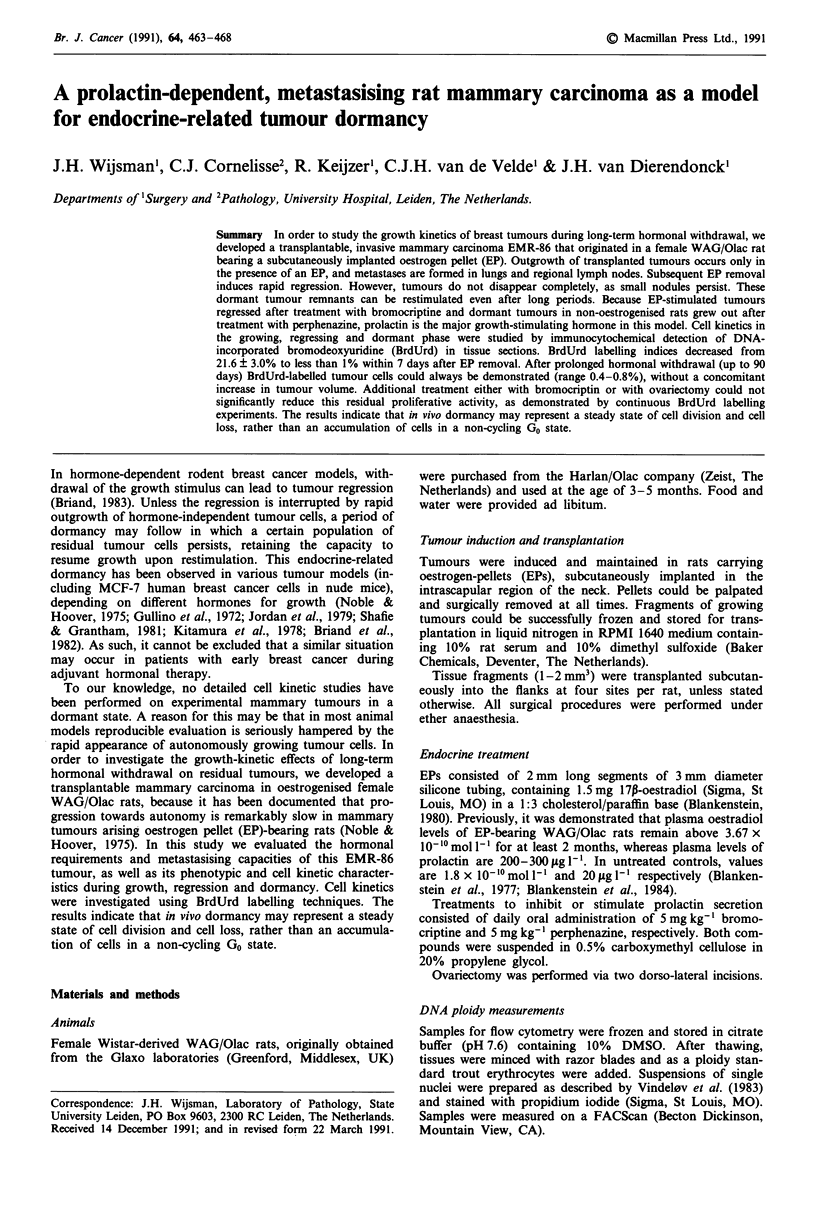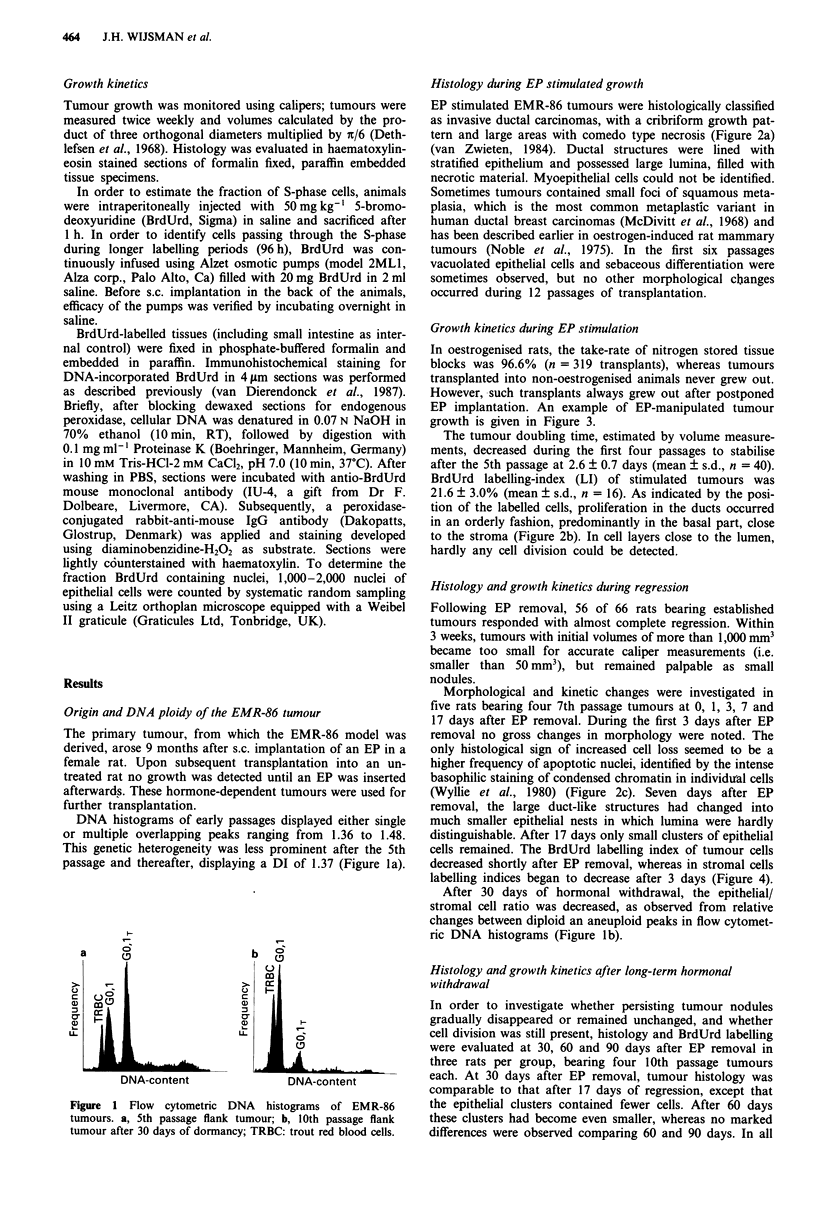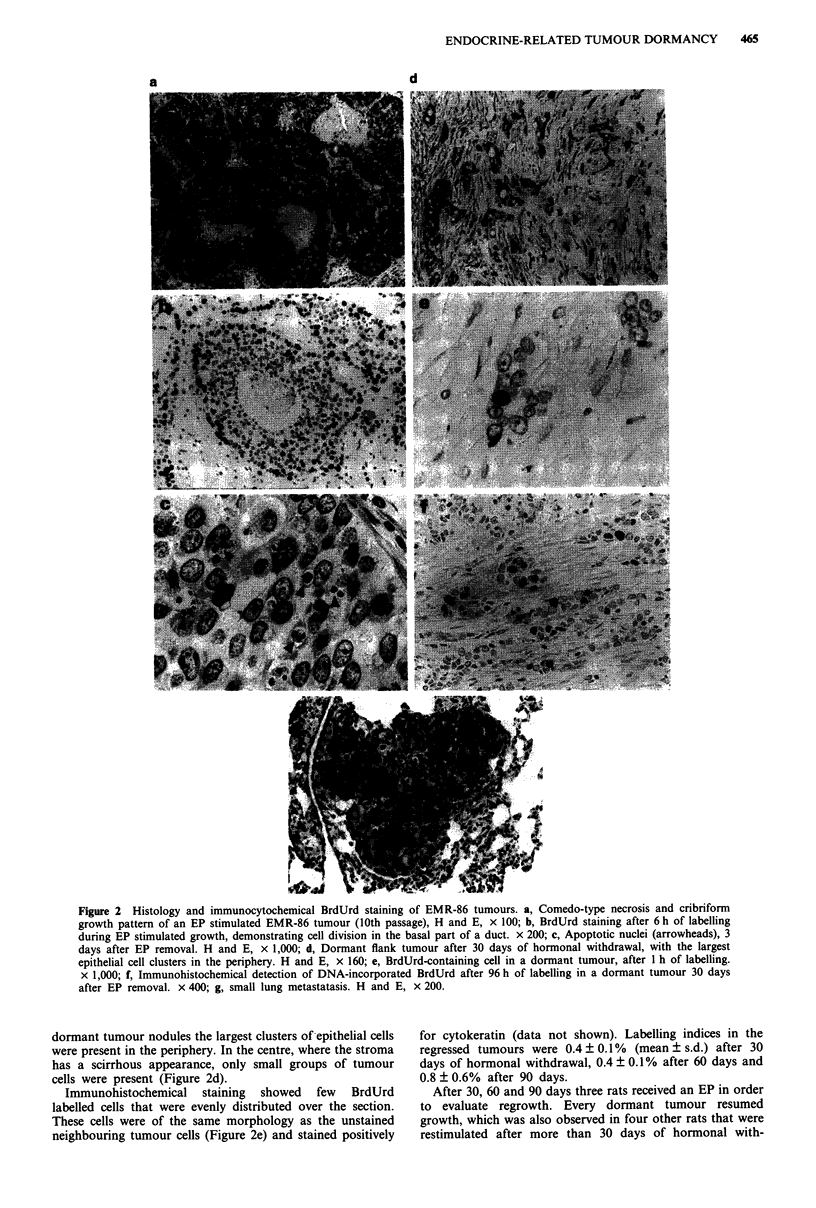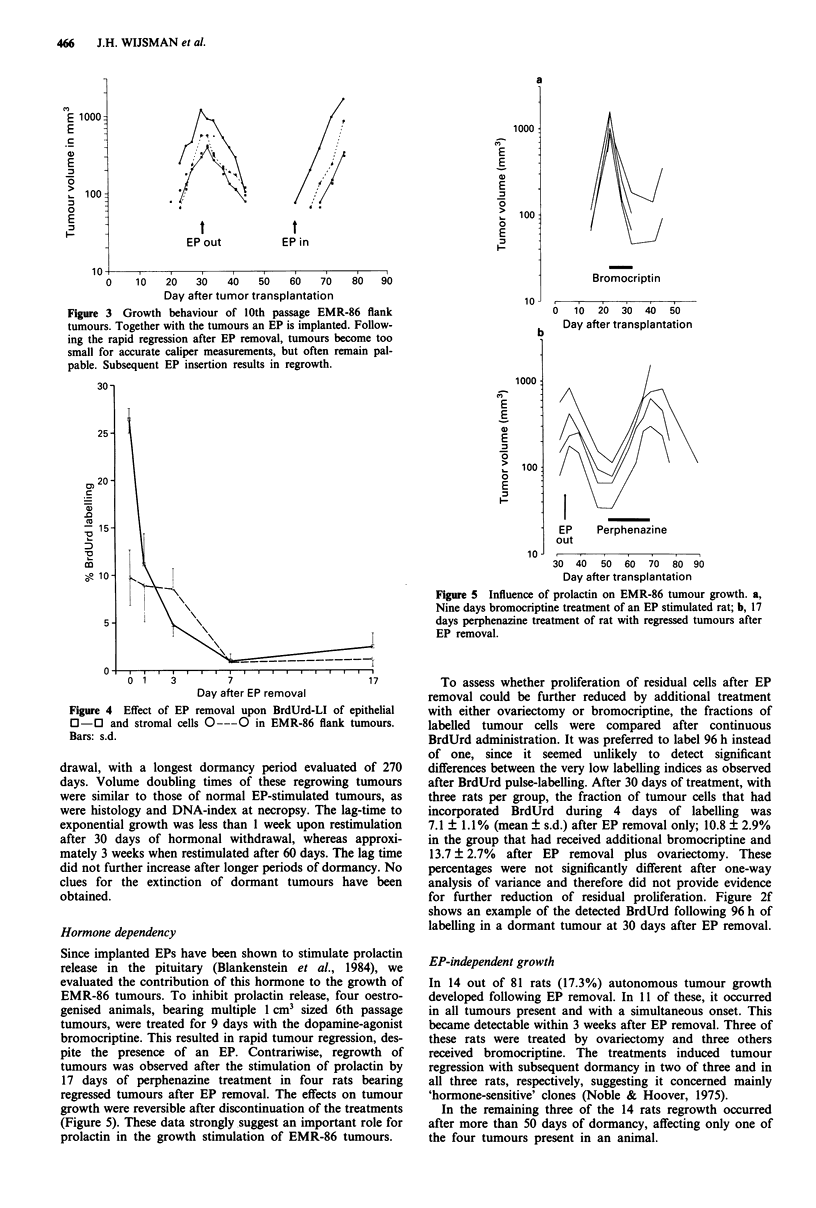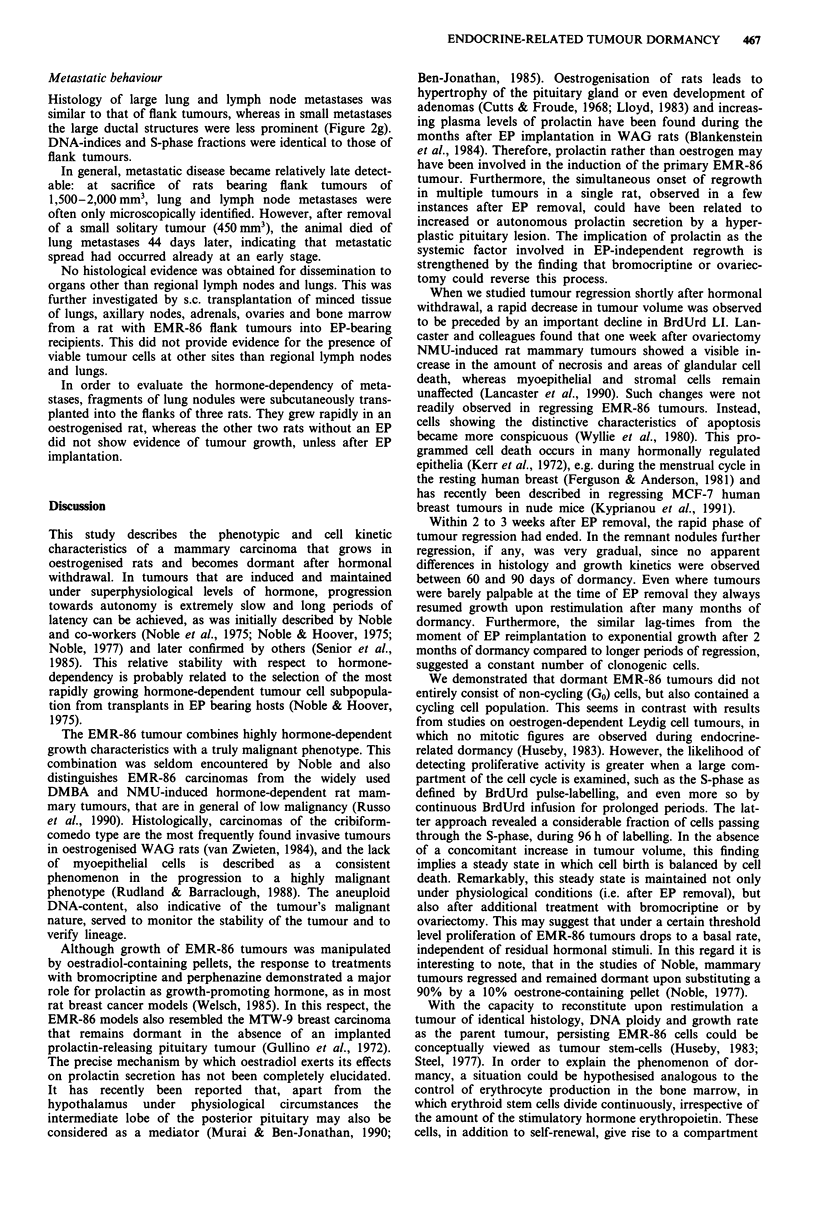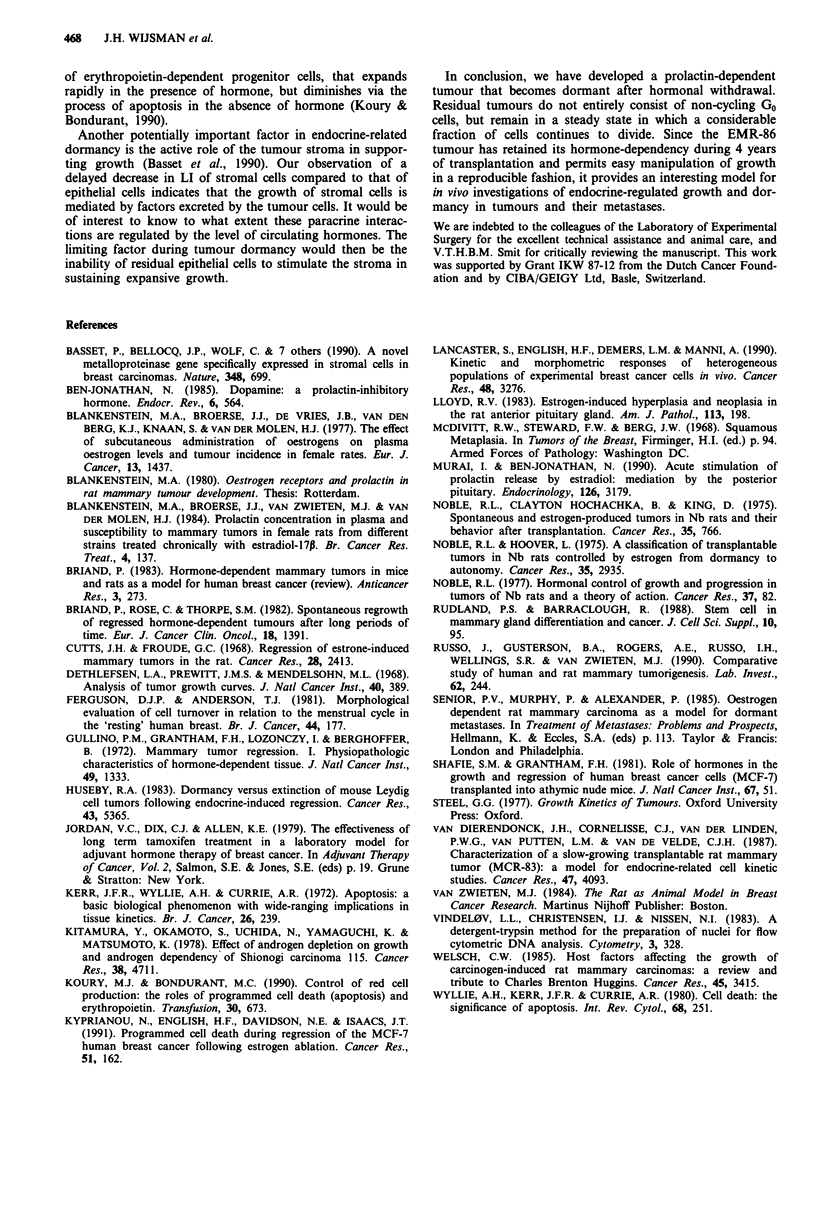# A prolactin-dependent, metastasising rat mammary carcinoma as a model for endocrine-related tumour dormancy.

**DOI:** 10.1038/bjc.1991.332

**Published:** 1991-09

**Authors:** J. H. Wijsman, C. J. Cornelisse, R. Keijzer, C. J. van de Velde, J. H. van Dierendonck

**Affiliations:** Department of Surgery, University Hospital, Leiden, The Netherlands.

## Abstract

**Images:**


Br. J. Cancer (1991), 64, 463-468 © Macmillan Press Ltd., 1991

### A prolactin-dependent, metastasising rat mammary carcinoma as a model for endocrine-related tumour dormancy

J.H. Wijsman[1], C.J. Cornelisse[2], R. Keijzer[1], C.J.H. van de Velde[1] & J.H. van Dierendonck[1]

*Departments of [1]Surgery and [2]Pathology, University Hospital, Leiden, The Netherlands.*

**Summary** In order to study the growth kinetics of breast tumours during long-term hormonal withdrawal, we developed a transplantable, invasive mammary carcinoma EMR-86 that originated in a female WAG/Olac rat bearing a subcutaneously implanted oestrogen pellet (EP). Outgrowth of transplanted tumours occurs only in the presence of an EP, and metastases are formed in lungs and regional lymph nodes. Subsequent EP removal induces rapid regression. However, tumours do not disappear completely, as small nodules persist. These dormant tumour remnants can be restimulated even after long periods. Because EP-stimulated tumours regressed after treatment with bromocriptine and dormant tumours in non-oestrogenised rats grew out after treatment with perphenazine, prolactin is the major growth-stimulating hormone in this model. Cell kinetics in the growing, regressing and dormant phase were studied by immunocytochemical detection of DNA-incorporated bromodeoxyuridine (BrdUrd) in tissue sections. BrdUrd labelling indices decreased from 21.6 ± 3.0% to less than 1% within 7 days after EP removal. After prolonged hormonal withdrawal (up to 90 days) BrdUrd-labelled tumour cells could always be demonstrated (range 0.4–0.8%), without a concomitant increase in tumour volume. Additional treatment either with bromocriptin or with ovariectomy could not significantly reduce this residual proliferative activity, as demonstrated by continuous BrdUrd labelling experiments. The results indicate that *in vivo* dormancy may represent a steady state of cell division and cell loss, rather than an accumulation of cells in a non-cycling $G_0$ state.

In hormone-dependent rodent breast cancer models, withdrawal of the growth stimulus can lead to tumour regression (Briand, 1983). Unless the regression is interrupted by rapid outgrowth of hormone-independent tumour cells, a period of dormancy may follow in which a certain population of residual tumour cells persists, retaining the capacity to resume growth upon restimulation. This endocrine-related dormancy has been observed in various tumour models (including MCF-7 human breast cancer cells in nude mice), depending on different hormones for growth (Noble & Hoover, 1975; Gullino *et al.*, 1972; Jordan *et al.*, 1979; Shafie & Grantham, 1981; Kitamura *et al.*, 1978; Briand *et al.*, 1982). As such, it cannot be excluded that a similar situation may occur in patients with early breast cancer during adjuvant hormonal therapy.

To our knowledge, no detailed cell kinetic studies have been performed on experimental mammary tumours in a dormant state. A reason for this may be that in most animal models reproducible evaluation is seriously hampered by the rapid appearance of autonomously growing tumour cells. In order to investigate the growth-kinetic effects of long-term hormonal withdrawal on residual tumours, we developed a transplantable mammary carcinoma in oestrogenised female WAG/Olac rats, because it has been documented that progression towards autonomy is remarkably slow in mammary tumours arising oestrogen pellet (EP)-bearing rats (Noble & Hoover, 1975). In this study we evaluated the hormonal requirements and metastasising capacities of this EMR-86 tumour, as well as its phenotypic and cell kinetic characteristics during growth, regression and dormancy. Cell kinetics were investigated using BrdUrd labelling techniques. The results indicate that *in vivo* dormancy may represent a steady state of cell division and cell loss, rather than an accumulation of cells in a non-cycling $G_0$ state.

### Materials and methods

### *Animals*

Female Wistar-derived WAG/Olac rats, originally obtained from the Glaxo laboratories (Greenford, Middlesex, UK) were purchased from the Harlan/Olac company (Zeist, The Netherlands) and used at the age of 3–5 months. Food and water were provided ad libitum.

### *Tumour induction and transplantation*

Tumours were induced and maintained in rats carrying oestrogen-pellets (EPs), subcutaneously implanted in the intrascapular region of the neck. Pellets could be palpated and surgically removed at all times. Fragments of growing tumours could be successfully frozen and stored for transplantation in liquid nitrogen in RPMI 1640 medium containing 10% rat serum and 10% dimethyl sulfoxide (Baker Chemicals, Deventer, The Netherlands).

Tissue fragments (1–2 $mm^3$) were transplanted subcutaneously into the flanks at four sites per rat, unless stated otherwise. All surgical procedures were performed under ether anaesthesia.

### *Endocrine treatment*

EPs consisted of 2 mm long segments of 3 mm diameter silicone tubing, containing 1.5 mg 17β-oestradiol (Sigma, St Louis, MO) in a 1:3 cholesterol/paraffin base (Blankenstein, 1980). Previously, it was demonstrated that plasma oestradiol levels of EP-bearing WAG/Olac rats remain above $3.67 \times 10^{-10}$ mol $l^{-1}$ for at least 2 months, whereas plasma levels of prolactin are 200–300 $\mu g\ l^{-1}$. In untreated controls, values are $1.8 \times 10^{-10}$ mol $l^{-1}$ and 20 $\mu g\ l^{-1}$ respectively (Blankenstein *et al.*, 1977; Blankenstein *et al.*, 1984).

Treatments to inhibit or stimulate prolactin secretion consisted of daily oral administration of 5 mg $kg^{-1}$ bromocriptine and 5 mg $kg^{-1}$ perphenazine, respectively. Both compounds were suspended in 0.5% carboxymethyl cellulose in 20% propylene glycol.

Ovariectomy was performed via two dorso-lateral incisions.

### *DNA ploidy measurements*

Samples for flow cytometry were frozen and stored in citrate buffer (pH 7.6) containing 10% DMSO. After thawing, tissues were minced with razor blades and as a ploidy standard trout erythrocytes were added. Suspensions of single nuclei were prepared as described by Vindeløv *et al.* (1983) and stained with propidium iodide (Sigma, St Louis, MO). Samples were measured on a FACScan (Becton Dickinson, Mountain View, CA).

Correspondence: J.H. Wijsman, Laboratory of Pathology, State University Leiden, PO Box 9603, 2300 RC Leiden, The Netherlands.
Received 14 December 1991; and in revised form 22 March 1991.

*Growth kinetics*

Tumour growth was monitored using calipers; tumours were measured twice weekly and volumes calculated by the product of three orthogonal diameters multiplied by $\pi/6$ (Dethlefsen *et al.*, 1968). Histology was evaluated in haematoxylin-eosin stained sections of formalin fixed, paraffin embedded tissue specimens.

In order to estimate the fraction of S-phase cells, animals were intraperitoneally injected with $50\ mg\ kg^{-1}$ 5-bromodeoxyuridine (BrdUrd, Sigma) in saline and sacrificed after 1 h. In order to identify cells passing through the S-phase during longer labelling periods (96 h), BrdUrd was continuously infused using Alzet osmotic pumps (model 2ML1, Alza corp., Palo Alto, Ca) filled with 20 mg BrdUrd in 2 ml saline. Before s.c. implantation in the back of the animals, efficacy of the pumps was verified by incubating overnight in saline.

BrdUrd-labelled tissues (including small intestine as internal control) were fixed in phosphate-buffered formalin and embedded in paraffin. Immunohistochemical staining for DNA-incorporated BrdUrd in 4 $\mu$m sections was performed as described previously (van Dierendonck *et al.*, 1987). Briefly, after blocking dewaxed sections for endogenous peroxidase, cellular DNA was denatured in 0.07 N NaOH in 70% ethanol (10 min, RT), followed by digestion with $0.1\ mg\ ml^{-1}$ Proteinase K (Boehringer, Mannheim, Germany) in 10 mM Tris-HCl-2 mM $CaCl_2$, pH 7.0 (10 min, 37°C). After washing in PBS, sections were incubated with antio-BrdUrd mouse monoclonal antibody (IU-4, a gift from Dr F. Dolbeare, Livermore, CA). Subsequently, a peroxidase-conjugated rabbit-anti-mouse IgG antibody (Dakopatts, Glostrup, Denmark) was applied and staining developed using diaminobenzidine-$H_2O_2$ as substrate. Sections were lightly counterstained with haematoxylin. To determine the fraction BrdUrd containing nuclei, 1,000–2,000 nuclei of epithelial cells were counted by systematic random sampling using a Leitz orthoplan microscope equipped with a Weibel II graticule (Graticules Ltd, Tonbridge, UK).

### Results

*Origin and DNA ploidy of the EMR-86 tumour*

The primary tumour, from which the EMR-86 model was derived, arose 9 months after s.c. implantation of an EP in a female rat. Upon subsequent transplantation into an untreated rat no growth was detected until an EP was inserted afterwards. These hormone-dependent tumours were used for further transplantation.

DNA histograms of early passages displayed either single or multiple overlapping peaks ranging from 1.36 to 1.48. This genetic heterogeneity was less prominent after the 5th passage and thereafter, displaying a DI of 1.37 (Figure 1a).

**Figure 1** Flow cytometric DNA histograms of EMR-86 tumours. **a**, 5th passage flank tumour; **b**, 10th passage flank tumour after 30 days of dormancy; TRBC: trout red blood cells.

*Histology during EP stimulated growth*

EP stimulated EMR-86 tumours were histologically classified as invasive ductal carcinomas, with a cribriform growth pattern and large areas with comedo type necrosis (Figure 2a) (van Zwieten, 1984). Ductal structures were lined with stratified epithelium and possessed large lumina, filled with necrotic material. Myoepithelial cells could not be identified. Sometimes tumours contained small foci of squamous metaplasia, which is the most common metaplastic variant in human ductal breast carcinomas (McDivitt *et al.*, 1968) and has been described earlier in oestrogen-induced rat mammary tumours (Noble *et al.*, 1975). In the first six passages vacuolated epithelial cells and sebaceous differentiation were sometimes observed, but no other morphological changes occurred during 12 passages of transplantation.

*Growth kinetics during EP stimulation*

In oestrogenised rats, the take-rate of nitrogen stored tissue blocks was 96.6% ($n = 319$ transplants), whereas tumours transplanted into non-oestrogenised animals never grew out. However, such transplants always grew out after postponed EP implantation. An example of EP-manipulated tumour growth is given in Figure 3.

The tumour doubling time, estimated by volume measurements, decreased during the first four passages to stabilise after the 5th passage at $2.6 \pm 0.7$ days (mean $\pm$ s.d., $n = 40$). BrdUrd labelling-index (LI) of stimulated tumours was $21.6 \pm 3.0\%$ (mean $\pm$ s.d., $n = 16$). As indicated by the position of the labelled cells, proliferation in the ducts occurred in an orderly fashion, predominantly in the basal part, close to the stroma (Figure 2b). In cell layers close to the lumen, hardly any cell division could be detected.

*Histology and growth kinetics during regression*

Following EP removal, 56 of 66 rats bearing established tumours responded with almost complete regression. Within 3 weeks, tumours with initial volumes of more than $1,000\ mm^3$ became too small for accurate caliper measurements (i.e. smaller than $50\ mm^3$), but remained palpable as small nodules.

Morphological and kinetic changes were investigated in five rats bearing four 7th passage tumours at 0, 1, 3, 7 and 17 days after EP removal. During the first 3 days after EP removal no gross changes in morphology were noted. The only histological sign of increased cell loss seemed to be a higher frequency of apoptotic nuclei, identified by the intense basophilic staining of condensed chromatin in individual cells (Wyllie *et al.*, 1980) (Figure 2c). Seven days after EP removal, the large duct-like structures had changed into much smaller epithelial nests in which lumina were hardly distinguishable. After 17 days only small clusters of epithelial cells remained. The BrdUrd labelling index of tumour cells decreased shortly after EP removal, whereas in stromal cells labelling indices began to decrease after 3 days (Figure 4).

After 30 days of hormonal withdrawal, the epithelial/stromal cell ratio was decreased, as observed from relative changes between diploid an aneuploid peaks in flow cytometric DNA histograms (Figure 1b).

*Histology and growth kinetics after long-term hormonal withdrawal*

In order to investigate whether persisting tumour nodules gradually disappeared or remained unchanged, and whether cell division was still present, histology and BrdUrd labelling were evaluated at 30, 60 and 90 days after EP removal in three rats per group, bearing four 10th passage tumours each. At 30 days after EP removal, tumour histology was comparable to that after 17 days of regression, except that the epithelial clusters contained fewer cells. After 60 days these clusters had become even smaller, whereas no marked differences were observed comparing 60 and 90 days. In all

**Figure 2** Histology and immunocytochemical BrdUrd staining of EMR-86 tumours. **a**, Comedo-type necrosis and cribriform growth pattern of an EP stimulated EMR-86 tumour (10th passage), H and E, × 100; **b**, BrdUrd staining after 6 h of labelling during EP stimulated growth, demonstrating cell division in the basal part of a duct. × 200; **c**, Apoptotic nuclei (arrowheads), 3 days after EP removal. H and E, × 1,000; **d**, Dormant flank tumour after 30 days of hormonal withdrawal, with the largest epithelial cell clusters in the periphery. H and E, × 160; **e**, BrdUrd-containing cell in a dormant tumour, after 1 h of labelling. × 1,000; **f**, Immunohistochemical detection of DNA-incorporated BrdUrd after 96 h of labelling in a dormant tumour 30 days after EP removal. × 400; **g**, small lung metastatasis. H and E, × 200.

dormant tumour nodules the largest clusters of epithelial cells were present in the periphery. In the centre, where the stroma has a scirrhous appearance, only small groups of tumour cells were present (Figure 2d).

Immunohistochemical staining showed few BrdUrd labelled cells that were evenly distributed over the section. These cells were of the same morphology as the unstained neighbouring tumour cells (Figure 2e) and stained positively for cytokeratin (data not shown). Labelling indices in the regressed tumours were 0.4 ± 0.1% (mean ± s.d.) after 30 days of hormonal withdrawal, 0.4 ± 0.1% after 60 days and 0.8 ± 0.6% after 90 days.

After 30, 60 and 90 days three rats received an EP in order to evaluate regrowth. Every dormant tumour resumed growth, which was also observed in four other rats that were restimulated after more than 30 days of hormonal with-

**Figure 3** Growth behaviour of 10th passage EMR-86 flank tumours. Together with the tumours an EP is implanted. Following the rapid regression after EP removal, tumours become too small for accurate caliper measurements, but often remain palpable. Subsequent EP insertion results in regrowth.

**Figure 4** Effect of EP removal upon BrdUrd-LI of epithelial □—□ and stromal cells O---O in EMR-86 flank tumours. Bars: s.d.

**Figure 5** Influence of prolactin on EMR-86 tumour growth. **a**, Nine days bromocriptine treatment of an EP stimulated rat; **b**, 17 days perphenazine treatment of rat with regressed tumours after EP removal.

drawal, with a longest dormancy period evaluated of 270 days. Volume doubling times of these regrowing tumours were similar to those of normal EP-stimulated tumours, as were histology and DNA-index at necropsy. The lag-time to exponential growth was less than 1 week upon restimulation after 30 days of hormonal withdrawal, whereas approximately 3 weeks when restimulated after 60 days. The lag time did not further increase after longer periods of dormancy. No clues for the extinction of dormant tumours have been obtained.

*Hormone dependency*

Since implanted EPs have been shown to stimulate prolactin release in the pituitary (Blankenstein *et al.*, 1984), we evaluated the contribution of this hormone to the growth of EMR-86 tumours. To inhibit prolactin release, four oestrogenised animals, bearing multiple 1 $cm^3$ sized 6th passage tumours, were treated for 9 days with the dopamine-agonist bromocriptine. This resulted in rapid tumour regression, despite the presence of an EP. Contrariwise, regrowth of tumours was observed after the stimulation of prolactin by 17 days of perphenazine treatment in four rats bearing regressed tumours after EP removal. The effects on tumour growth were reversible after discontinuation of the treatments (Figure 5). These data strongly suggest an important role for prolactin in the growth stimulation of EMR-86 tumours.

To assess whether proliferation of residual cells after EP removal could be further reduced by additional treatment with either ovariectomy or bromocriptine, the fractions of labelled tumour cells were compared after continuous BrdUrd administration. It was preferred to label 96 h instead of one, since it seemed unlikely to detect significant differences between the very low labelling indices as observed after BrdUrd pulse-labelling. After 30 days of treatment, with three rats per group, the fraction of tumour cells that had incorporated BrdUrd during 4 days of labelling was $7.1 \pm 1.1\%$ (mean ± s.d.) after EP removal only; $10.8 \pm 2.9\%$ in the group that had received additional bromocriptine and $13.7 \pm 2.7\%$ after EP removal plus ovariectomy. These percentages were not significantly different after one-way analysis of variance and therefore did not provide evidence for further reduction of residual proliferation. Figure 2f shows an example of the detected BrdUrd following 96 h of labelling in a dormant tumour at 30 days after EP removal.

*EP-independent growth*

In 14 out of 81 rats (17.3%) autonomous tumour growth developed following EP removal. In 11 of these, it occurred in all tumours present and with a simultaneous onset. This became detectable within 3 weeks after EP removal. Three of these rats were treated by ovariectomy and three others received bromocriptine. The treatments induced tumour regression with subsequent dormancy in two of three and in all three rats, respectively, suggesting it concerned mainly 'hormone-sensitive' clones (Noble & Hoover, 1975).

In the remaining three of the 14 rats regrowth occurred after more than 50 days of dormancy, affecting only one of the four tumours present in an animal.

*Metastatic behaviour*

Histology of large lung and lymph node metastases was similar to that of flank tumours, whereas in small metastases the large ductal structures were less prominent (Figure 2g). DNA-indices and S-phase fractions were identical to those of flank tumours.

In general, metastatic disease became relatively late detectable: at sacrifice of rats bearing flank tumours of 1,500–2,000 $mm^3$, lung and lymph node metastases were often only microscopically identified. However, after removal of a small solitary tumour (450 $mm^3$), the animal died of lung metastases 44 days later, indicating that metastatic spread had occurred already at an early stage.

No histological evidence was obtained for dissemination to organs other than regional lymph nodes and lungs. This was further investigated by s.c. transplantation of minced tissue of lungs, axillary nodes, adrenals, ovaries and bone marrow from a rat with EMR-86 flank tumours into EP-bearing recipients. This did not provide evidence for the presence of viable tumour cells at other sites than regional lymph nodes and lungs.

In order to evaluate the hormone-dependency of metastases, fragments of lung nodules were subcutaneously transplanted into the flanks of three rats. They grew rapidly in an oestrogenised rat, whereas the other two rats without an EP did not show evidence of tumour growth, unless after EP implantation.

### Discussion

This study describes the phenotypic and cell kinetic characteristics of a mammary carcinoma that grows in oestrogenised rats and becomes dormant after hormonal withdrawal. In tumours that are induced and maintained under superphysiological levels of hormone, progression towards autonomy is extremely slow and long periods of latency can be achieved, as was initially described by Noble and co-workers (Noble *et al.*, 1975; Noble & Hoover, 1975; Noble, 1977) and later confirmed by others (Senior *et al.*, 1985). This relative stability with respect to hormone-dependency is probably related to the selection of the most rapidly growing hormone-dependent tumour cell subpopulation from transplants in EP bearing hosts (Noble & Hoover, 1975).

The EMR-86 tumour combines highly hormone-dependent growth characteristics with a truly malignant phenotype. This combination was seldom encountered by Noble and also distinguishes EMR-86 carcinomas from the widely used DMBA and NMU-induced hormone-dependent rat mammary tumours, that are in general of low malignancy (Russo *et al.*, 1990). Histologically, carcinomas of the cribiform-comedo type are the most frequently found invasive tumours in oestrogenised WAG rats (van Zwieten, 1984), and the lack of myoepithelial cells is described as a consistent phenomenon in the progression to a highly malignant phenotype (Rudland & Barraclough, 1988). The aneuploid DNA-content, also indicative of the tumour's malignant nature, served to monitor the stability of the tumour and to verify lineage.

Although growth of EMR-86 tumours was manipulated by oestradiol-containing pellets, the response to treatments with bromocriptine and perphenazine demonstrated a major role for prolactin as growth-promoting hormone, as in most rat breast cancer models (Welsch, 1985). In this respect, the EMR-86 models also resembled the MTW-9 breast carcinoma that remains dormant in the absence of an implanted prolactin-releasing pituitary tumour (Gullino *et al.*, 1972). The precise mechanism by which oestradiol exerts its effects on prolactin secretion has not been completely elucidated. It has recently been reported that, apart from the hypothalamus under physiological circumstances the intermediate lobe of the posterior pituitary may also be considered as a mediator (Murai & Ben-Jonathan, 1990; Ben-Jonathan, 1985). Oestrogenisation of rats leads to hypertrophy of the pituitary gland or even development of adenomas (Cutts & Froude, 1968; Lloyd, 1983) and increasing plasma levels of prolactin have been found during the months after EP implantation in WAG rats (Blankenstein *et al.*, 1984). Therefore, prolactin rather than oestrogen may have been involved in the induction of the primary EMR-86 tumour. Furthermore, the simultaneous onset of regrowth in multiple tumours in a single rat, observed in a few instances after EP removal, could have been related to increased or autonomous prolactin secretion by a hyperplastic pituitary lesion. The implication of prolactin as the systemic factor involved in EP-independent regrowth is strengthened by the finding that bromocriptine or ovariectomy could reverse this process.

When we studied tumour regression shortly after hormonal withdrawal, a rapid decrease in tumour volume was observed to be preceded by an important decline in BrdUrd LI. Lancaster and colleagues found that one week after ovariectomy NMU-induced rat mammary tumours showed a visible increase in the amount of necrosis and areas of glandular cell death, whereas myoepithelial and stromal cells remain unaffected (Lancaster *et al.*, 1990). Such changes were not readily observed in regressing EMR-86 tumours. Instead, cells showing the distinctive characteristics of apoptosis became more conspicuous (Wyllie *et al.*, 1980). This programmed cell death occurs in many hormonally regulated epithelia (Kerr *et al.*, 1972), e.g. during the menstrual cycle in the resting human breast (Ferguson & Anderson, 1981) and has recently been described in regressing MCF-7 human breast tumours in nude mice (Kyprianou *et al.*, 1991).

Within 2 to 3 weeks after EP removal, the rapid phase of tumour regression had ended. In the remnant nodules further regression, if any, was very gradual, since no apparent differences in histology and growth kinetics were observed between 60 and 90 days of dormancy. Even where tumours were barely palpable at the time of EP removal they always resumed growth upon restimulation after many months of dormancy. Furthermore, the similar lag-times from the moment of EP reimplantation to exponential growth after 2 months of dormancy compared to longer periods of regression, suggested a constant number of clonogenic cells.

We demonstrated that dormant EMR-86 tumours did not entirely consist of non-cycling ($G_0$) cells, but also contained a cycling cell population. This seems in contrast with results from studies on oestrogen-dependent Leydig cell tumours, in which no mitotic figures are observed during endocrine-related dormancy (Huseby, 1983). However, the likelihood of detecting proliferative activity is greater when a large compartment of the cell cycle is examined, such as the S-phase as defined by BrdUrd pulse-labelling, and even more so by continuous BrdUrd infusion for prolonged periods. The latter approach revealed a considerable fraction of cells passing through the S-phase, during 96 h of labelling. In the absence of a concomitant increase in tumour volume, this finding implies a steady state in which cell birth is balanced by cell death. Remarkably, this steady state is maintained not only under physiological conditions (i.e. after EP removal), but also after additional treatment with bromocriptine or by ovariectomy. This may suggest that under a certain threshold level proliferation of EMR-86 tumours drops to a basal rate, independent of residual hormonal stimuli. In this regard it is interesting to note, that in the studies of Noble, mammary tumours regressed and remained dormant upon substituting a 90% by a 10% oestrone-containing pellet (Noble, 1977).

With the capacity to reconstitute upon restimulation a tumour of identical histology, DNA ploidy and growth rate as the parent tumour, persisting EMR-86 cells could be conceptually viewed as tumour stem-cells (Huseby, 1983; Steel, 1977). In order to explain the phenomenon of dormancy, a situation could be hypothesised analogous to the control of erythrocyte production in the bone marrow, in which erythroid stem cells divide continuously, irrespective of the amount of the stimulatory hormone erythropoietin. These cells, in addition to self-renewal, give rise to a compartment of erythropoietin-dependent progenitor cells, that expands rapidly in the presence of hormone, but diminishes via the process of apoptosis in the absence of hormone (Koury & Bondurant, 1990).

Another potentially important factor in endocrine-related dormancy is the active role of the tumour stroma in supporting growth (Basset *et al.*, 1990). Our observation of a delayed decrease in LI of stromal cells compared to that of epithelial cells indicates that the growth of stromal cells is mediated by factors excreted by the tumour cells. It would be of interest to know to what extent these paracrine interactions are regulated by the level of circulating hormones. The limiting factor during tumour dormancy would then be the inability of residual epithelial cells to stimulate the stroma in sustaining expansive growth.

In conclusion, we have developed a prolactin-dependent tumour that becomes dormant after hormonal withdrawal. Residual tumours do not entirely consist of non-cycling $G_0$ cells, but remain in a steady state in which a considerable fraction of cells continues to divide. Since the EMR-86 tumour has retained its hormone-dependency during 4 years of transplantation and permits easy manipulation of growth in a reproducible fashion, it provides an interesting model for *in vivo* investigations of endocrine-regulated growth and dormancy in tumours and their metastases.

We are indebted to the colleagues of the Laboratory of Experimental Surgery for the excellent technical assistance and animal care, and V.T.H.B.M. Smit for critically reviewing the manuscript. This work was supported by Grant IKW 87-12 from the Dutch Cancer Foundation and by CIBA/GEIGY Ltd, Basle, Switzerland.

### References

BASSET, P., BELLOCQ, J.P., WOLF, C. & 7 others (1990). A novel metalloproteinase gene specifically expressed in stromal cells in breast carcinomas. *Nature*, **348**, 699.

BEN-JONATHAN, N. (1985). Dopamine: a prolactin-inhibitory hormone. *Endocr. Rev.*, **6**, 564.

BLANKENSTEIN, M.A., BROERSE, J.J., DE VRIES, J.B., VAN DEN BERG, K.J., KNAAN, S. & VAN DER MOLEN, H.J. (1977). The effect of subcutaneous administration of oestrogens on plasma oestrogen levels and tumour incidence in female rates. *Eur. J. Cancer*, **13**, 1437.

BLANKENSTEIN, M.A. (1980). *Oestrogen receptors and prolactin in rat mammary tumour development*. Thesis: Rotterdam.

BLANKENSTEIN, M.A., BROERSE, J.J., VAN ZWIETEN, M.J. & VAN DER MOLEN, H.J. (1984). Prolactin concentration in plasma and susceptibility to mammary tumors in female rats from different strains treated chronically with estradiol-17β. *Br. Cancer Res. Treat.*, **4**, 137.

BRIAND, P. (1983). Hormone-dependent mammary tumors in mice and rats as a model for human breast cancer (review). *Anticancer Res.*, **3**, 273.

BRIAND, P., ROSE, C. & THORPE, S.M. (1982). Spontaneous regrowth of regressed hormone-dependent tumours after long periods of time. *Eur. J. Cancer Clin. Oncol.*, **18**, 1391.

CUTTS, J.H. & FROUDE, G.C. (1968). Regression of estrone-induced mammary tumors in the rat. *Cancer Res.*, **28**, 2413.

DETHLEFSEN, L.A., PREWITT, J.M.S. & MENDELSOHN, M.L. (1968). Analysis of tumor growth curves. *J. Natl Cancer Inst.*, **40**, 389.

FERGUSON, D.J.P. & ANDERSON, T.J. (1981). Morphological evaluation of cell turnover in relation to the menstrual cycle in the 'resting' human breast. *Br. J. Cancer*, **44**, 177.

GULLINO, P.M., GRANTHAM, F.H., LOZONCZY, I. & BERGHOFFER, B. (1972). Mammary tumor regression. I. Physiopathologic characteristics of hormone-dependent tissue. *J. Natl Cancer Inst.*, **49**, 1333.

HUSEBY, R.A. (1983). Dormancy versus extinction of mouse Leydig cell tumors following endocrine-induced regression. *Cancer Res.*, **43**, 5365.

JORDAN, V.C., DIX, C.J. & ALLEN, K.E. (1979). The effectiveness of long term tamoxifen treatment in a laboratory model for adjuvant hormone therapy of breast cancer. In *Adjuvant Therapy of Cancer, Vol. 2*, Salmon, S.E. & Jones, S.E. (eds) p. 19. Grune & Stratton: New York.

KERR, J.F.R., WYLLIE, A.H. & CURRIE, A.R. (1972). Apoptosis: a basic biological phenomenon with wide-ranging implications in tissue kinetics. *Br. J. Cancer*, **26**, 239.

KITAMURA, Y., OKAMOTO, S., UCHIDA, N., YAMAGUCHI, K. & MATSUMOTO, K. (1978). Effect of androgen depletion on growth and androgen dependency of Shionogi carcinoma 115. *Cancer Res.*, **38**, 4711.

KOURY, M.J. & BONDURANT, M.C. (1990). Control of red cell production: the roles of programmed cell death (apoptosis) and erythropoietin. *Transfusion*, **30**, 673.

KYPRIANOU, N., ENGLISH, H.F., DAVIDSON, N.E. & ISAACS, J.T. (1991). Programmed cell death during regression of the MCF-7 human breast cancer following estrogen ablation. *Cancer Res.*, **51**, 162.

LANCASTER, S., ENGLISH, H.F., DEMERS, L.M. & MANNI, A. (1990). Kinetic and morphometric responses of heterogeneous populations of experimental breast cancer cells *in vivo*. *Cancer Res.*, **48**, 3276.

LLOYD, R.V. (1983). Estrogen-induced hyperplasia and neoplasia in the rat anterior pituitary gland. *Am. J. Pathol.*, **113**, 198.

MCDIVITT, R.W., STEWARD, F.W. & BERG, J.W. (1968). Squamous Metaplasia. In *Tumors of the Breast*, Firminger, H.I. (ed.) p. 94. Armed Forces of Pathology: Washington DC.

MURAI, I. & BEN-JONATHAN, N. (1990). Acute stimulation of prolactin release by estradiol: mediation by the posterior pituitary. *Endocrinology*, **126**, 3179.

NOBLE, R.L., CLAYTON HOCHACHKA, B. & KING, D. (1975). Spontaneous and estrogen-produced tumors in Nb rats and their behavior after transplantation. *Cancer Res.*, **35**, 766.

NOBLE, R.L. & HOOVER, L. (1975). A classification of transplantable tumors in Nb rats controlled by estrogen from dormancy to autonomy. *Cancer Res.*, **35**, 2935.

NOBLE, R.L. (1977). Hormonal control of growth and progression in tumors of Nb rats and a theory of action. *Cancer Res.*, **37**, 82.

RUDLAND, P.S. & BARRACLOUGH, R. (1988). Stem cell in mammary gland differentiation and cancer. *J. Cell Sci. Suppl.*, **10**, 95.

RUSSO, J., GUSTERSON, B.A., ROGERS, A.E., RUSSO, I.H., WELLINGS, S.R. & VAN ZWIETEN, M.J. (1990). Comparative study of human and rat mammary tumorigenesis. *Lab. Invest.*, **62**, 244.

SENIOR, P.V., MURPHY, P. & ALEXANDER, P. (1985). Oestrogen dependent rat mammary carcinoma as a model for dormant metastases. In *Treatment of Metastases: Problems and Prospects*, Hellmann, K. & Eccles, S.A. (eds) p. 113. Taylor & Francis: London and Philadelphia.

SHAFIE, S.M. & GRANTHAM, F.H. (1981). Role of hormones in the growth and regression of human breast cancer cells (MCF-7) transplanted into athymic nude mice. *J. Natl Cancer Inst.*, **67**, 51.

STEEL, G.G. (1977). *Growth Kinetics of Tumours*. Oxford University Press: Oxford.

VAN DIERENDONCK, J.H., CORNELISSE, C.J., VAN DER LINDEN, P.W.G., VAN PUTTEN, L.M. & VAN DE VELDE, C.J.H. (1987). Characterization of a slow-growing transplantable rat mammary tumor (MCR-83): a model for endocrine-related cell kinetic studies. *Cancer Res.*, **47**, 4093.

VAN ZWIETEN, M.J. (1984). *The Rat as Animal Model in Breast Cancer Research*. Martinus Nijhoff Publisher: Boston.

VINDELØV, L.L., CHRISTENSEN, I.J. & NISSEN, N.I. (1983). A detergent-trypsin method for the preparation of nuclei for flow cytometric DNA analysis. *Cytometry*, **3**, 328.

WELSCH, C.W. (1985). Host factors affecting the growth of carcinogen-induced rat mammary carcinomas: a review and tribute to Charles Brenton Huggins. *Cancer Res.*, **45**, 3415.

WYLLIE, A.H., KERR, J.F.R. & CURRIE, A.R. (1980). Cell death: the significance of apoptosis. *Int. Rev. Cytol.*, **68**, 251.